# Disruption in murine *Eml1* perturbs retinal lamination during early development

**DOI:** 10.1038/s41598-020-62373-5

**Published:** 2020-03-27

**Authors:** G. B. Collin, J. Won, M. P. Krebs, W. J. Hicks, J. R. Charette, J. K. Naggert, P. M. Nishina

**Affiliations:** The Jackson Laboratory, 600 Main Street, Bar Harbor, Maine 04609 USA

**Keywords:** Disease model, Mutation, Retina

## Abstract

During mammalian development, establishing functional neural networks in stratified tissues of the mammalian central nervous system depends upon the proper migration and positioning of neurons, a process known as lamination. In particular, the pseudostratified neuroepithelia of the retina and cerebrocortical ventricular zones provide a platform for progenitor cell proliferation and migration. Lamination defects in these tissues lead to mispositioned neurons, disrupted neuronal connections, and abnormal function. The molecular mechanisms necessary for proper lamination in these tissues are incompletely understood. Here, we identified a nonsense mutation in the *Eml1* gene in a novel murine model, *tvrm360*, displaying subcortical heterotopia, hydrocephalus and disorganization of retinal architecture. In the retina, *Eml1* disruption caused abnormal positioning of photoreceptor cell nuclei early in development. Upon maturation, these ectopic photoreceptors possessed cilia and formed synapses but failed to produce robust outer segments, implying a late defect in photoreceptor differentiation secondary to mislocalization. In addition, abnormal positioning of Müller cell bodies and bipolar cells was evident throughout the inner neuroblastic layer. Basal displacement of mitotic nuclei in the retinal neuroepithelium was observed in *tvrm360* mice at postnatal day 0. The abnormal positioning of retinal progenitor cells at birth and ectopic presence of photoreceptors and secondary neurons upon maturation suggest that EML1 functions early in eye development and is crucial for proper retinal lamination during cellular proliferation and development.

## Introduction

In the developing brain and retina, the ventricular zone is a specialized pseudostratified epithelium that contains multi-potent neural progenitor cells^1^. Establishing apical-basal polarity and proper mitotic progenitor positioning at the ventricular surface is essential for neuronal lamination and homeostasis of the neuroepithelium^2^. The formation of layered neuronal populations, or lamination, in the mammalian central nervous system requires highly coordinated cellular movements that determine the final position of each cell within the tissue. In the cerebral cortex, radial glia provide an efficient framework for the guided movement of progenitor cells and post-mitotic neurons to form highly specialized cellular layers^3^. This process is assisted by interkinetic nuclear migration^4^ where proliferating neuroepithelial progenitor nuclei migrate between apical and basal surfaces in coordination with the cell cycle. Similarly, in the developing retina, nuclei migrate along microtubules synchronously with the cell cycle within the extended cytoplasm of retinal progenitor cells that populate the pseudostratified neuroepithelium^5–7^.

Disruption of the laminar organization in the developing central nervous system has been implicated in cerebral cortical disorders such as lissencephaly, subcortical band heterotopia, and periventricular nodular heterotopia^8^. Each of these conditions result from a failure of early post-mitotic neurons to migrate from the ventricular zone to the cortical plate. A subset of these disorders arise from migration defects in apical progenitor cells. In particular, lissencephaly-causing mutations result in inhibition of neuronal migration and ectopic mitotic figures throughout the ventricular zone of the cerebral cortex^9,10^. Studies in *Flna* knockdown rats, a model for periventricular nodular heterotopia, have shown abnormal cell cycle progression and neuronal migration as the cause for the disorganization in the ventricular zone radial glial scaffold^11^. In the retina, deficits in interkinetic nuclear migration during cell division have been shown to influence retinal architecture during neuroepithelial development^12–14^.

In humans, disruption of *EML1* which encodes echinoderm microtubule-associated protein like 1, is associated with defects in the developing brain that leads to bilateral giant ribbon-like subcortical heterotopia and hydrocephalus^15,16^. The primary defect appears to be due to ectopic apical progenitors distributed beyond the cortical proliferative zones and is unlike other forms of heterotopia that typically present with abnormalities in neuronal migration^15,17^.

In this report, we describe a novel allele of murine *Eml1, tvrm360*, bearing a nonsense mutation generated by chemical mutagenesis in the Translational Vision Research Models (TVRM) program at The Jackson Laboratory^18,19^. Similar to a previously described *HeCo* mouse model^17^, we show that *tvrm360* mutants develop bilateral subcortical heterotopia and hydrocephalus. In addition, we demonstrate new findings of retinal lamination defects in *Eml1*^*tvrm360*^ resulting in impaired photoreceptor function. Disruptions in retinal architecture are observed early in development and likely due to the improper positioning of mitotic apical progenitor nuclei during the cell cycle. *Eml1*^*tvrm360*^ mice will be a useful model for studying neuroblastic cell proliferation and microtubular dynamics in the developing retina.

## Results

### A point mutation in the gene for echinoderm microtubule associated protein 1, *Eml1*, leads to a premature stop codon

The TVRM program at The Jackson Laboratory identified an ENU-derived mouse model, *tvrm64*, which presented with a grainy appearing fundus that harbored a mutation in the retinitis pigmentosa 1 gene (*Rp1*)^18^, designated, C57BL/6J-*Rp1*^*tvrm64*^/PjnMmjax (MMRRC 043579). However, during the maintenance of the colony, it was noted that some mice developed severe hydrocephalus and died prematurely, shortly after weaning. By selecting against the *Rp1*^*tvrm64*^ allele in a series of backcrosses to strain C57BL/6J and subsequent intercrosses, we were able to segregate the *Rp1*^*tvrm64*^ fundus phenotype observable by indirect ophthalmoscopy from the hydrocephalus phenotype, confirming the existence of a second recessive mutation, which was subsequently named *tvrm360*.

The causative mutation in *tvrm360* was localized to Chromosome 12 in the *Rp1*^*tvrm64*^ mapping intercross using an affecteds-only mapping strategy scoring for the presence of hydrocephaly. Subsequently, 345 meioses were examined for recombinations in the interval between markers *D12Mit99* and *D12Mit262* (Fig. S1a). Fine mapping with additional markers using the recombinant DNAs of phenotyped mice, identified a 1.3 Mb critical region between markers *D12Mit233* and *rs29212532* that included 14 protein-coding transcripts (Table S1). A phenotypic driven query in the Mouse Genomic Informatics (MGI) database revealed two candidate genes: *Ccdc85c*, encoding coil-coil domain containing 85C, and *Eml1*, encoding echinoderm microtubule associated protein like 1. Previous reports indicated that disruption of either gene results in cerebral features such as heterotopia and hydrocephalus^15,20^. One recombination identified from the fine mapping cross was positioned within *Ccdc85c*. Sequencing of *Ccdc85c* transcripts downstream of the recombination did not reveal any sequence differences between C57BL/6J and *tvrm360*. However, sequencing of *Eml1* identified a transversion of nucleotide 1956 from T to A in exon 18 (Fig. S1b). The *Eml1*^*tvrm360*^ mutation is predicted to result in a premature termination of the EML1 polypeptide, p.Tyr652*, truncating a substantial portion of the C-terminal propeller domain of the protein (Fig. S1c).

**Figure 1:**
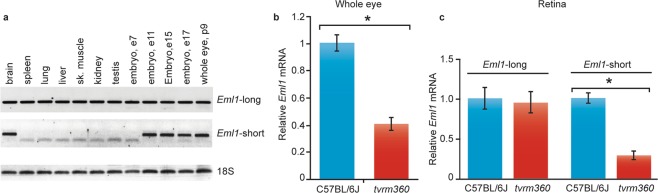
*Eml1* mRNA expression. (**a**) Expression of the long and short forms of *Eml1* transcriptional variants, as detected by RT-PCR, indicates differential expression across body tissues and during embryonic development. RT-PCR of 18 S RNA served as a loading control. (**b**) Quantitative RT-PCR of all three *Eml1* variants in *Eml1*^*tvrm360*^ (*tvrm360*) mutants (n = 5) and C57BL/6J control whole eyes (n = 5) at postnatal day 14. Student’s t-test; *P < 0.0001. (**c**) Quantitative PCR of retina at postnatal day 14 was performed using primers specific to the long and short forms of *Eml1* (n = 4 each group). Student’s t-test; *P < 0.05. Relative *Eml1* mRNA levels ± standard error are shown in b,c. B6J values are shown in blue and *Eml1*^*tvrm360*^ values are shown in red.

A search in the mouse Ensembl genome browser^21^ identified three predicted *Eml1* mRNA splice forms of EML1: *Eml1-203* (ENSMUST00000109860.7), a long form that encodes a polypeptide of 814 amino acids (aa) and two shorter forms, *Eml1-202* (ENSMUST00000109857.7) and *Eml1-201* (ENSMUST00000054955.13), that encode polypeptides of 800 and 783 aa in length. We examined the relative abundance of the *Eml1* transcripts during development by RT-PCR using exon1-specific primers that differentiate between the long form and the two shorter forms. The long form mRNA appears to be ubiquitously expressed during development (Fig. 1a) whereas the shorter forms are temporally and spatially restricted (after E11 during embryonic development, and in adult brain, and postnatal day 9 eye).

Comparison of quantitative RT–PCR levels of *Eml1*^*tvrm360*^ and WT control mRNA revealed a significant 2.5-fold reduction of *Eml1* mRNA in *tvrm360* whole eyes compared to WT mice (relative normalized expression; two tailed t-test; *p* < 0.0001) (Fig. 1b). This result suggests that some *Eml1* transcripts in *Eml1*^*tvrm360*^ ocular tissue undergo nonsense-mediated decay, a common observance in genetic diseases in which mutations result in premature translation termination^22^.

To determine which of the variants were specific to the neural retina, we performed quantitative PCR on *Eml1*^*tvrm360*^ and B6J control retinas at postnatal day 14. Both long and short form(s) were detected in the retinas of B6J controls, while only expression of the short form(s) was significantly reduced in *Eml1*^*tvrm360*^ retinas compared to controls (Fig. 1c).

### The *Eml1*^*tvrm360*^ mutation causes subcortical heterotopia and hydrocephaly

**Figure 2:**
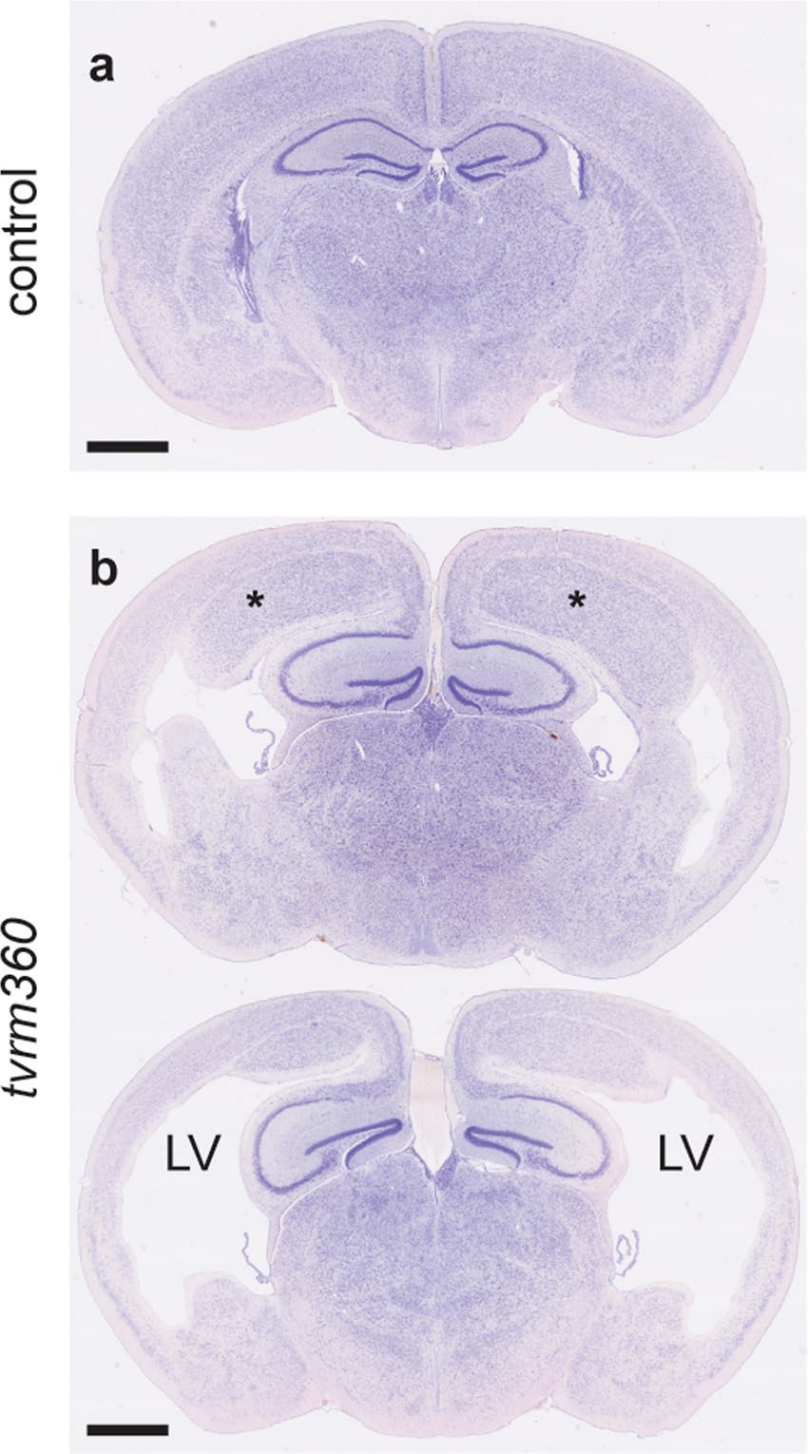
Subcortical heterotopia and hydrocephalus in homozygous *Eml1*^*tvrm360*^ mice. Coronal brain sections of wildtype (**a**) and mutant (**b**) mice stained with cresyl violet. Regions of nodular bilateral heterotopia (asterisk) and severe enlargement of the lateral ventricles (LV) were observed in *Eml1*^*tvrm360*^ mutants at one month (1 M) of age. Scale bar: 1 mm.

In our model, homozygous *Eml1*^*tvrm360*^ mutants develop hydrocephalus that is detectable between 1–2 weeks of birth with a bulging fontanelle and enlargement of the head. Most *Eml1*^*tvrm360*^ mutant mice die prior to weaning at three weeks of age with a few surviving to ~6 weeks of age, and in very rare instances, to 3 months of age. Presumably death results from the severe accumulation of cerebrospinal fluid (CSF) in the brain. Cresyl violet staining of coronal brain sections reveal dilated lateral ventricles and bilateral nodular heterotopia in the subcortical region (Fig. 2). This observation is akin to a previous report of mice bearing a spontaneous transposon insertion in *Eml1* (*Eml1*^*heco*^), which developed subcortical band heterotopia with occasional enlargement of lateral ventricles^17^.

### The *Eml1*^*tvrm360*^ mutation leads to gross morphological lamination defects in the retina

**Figure 3:**
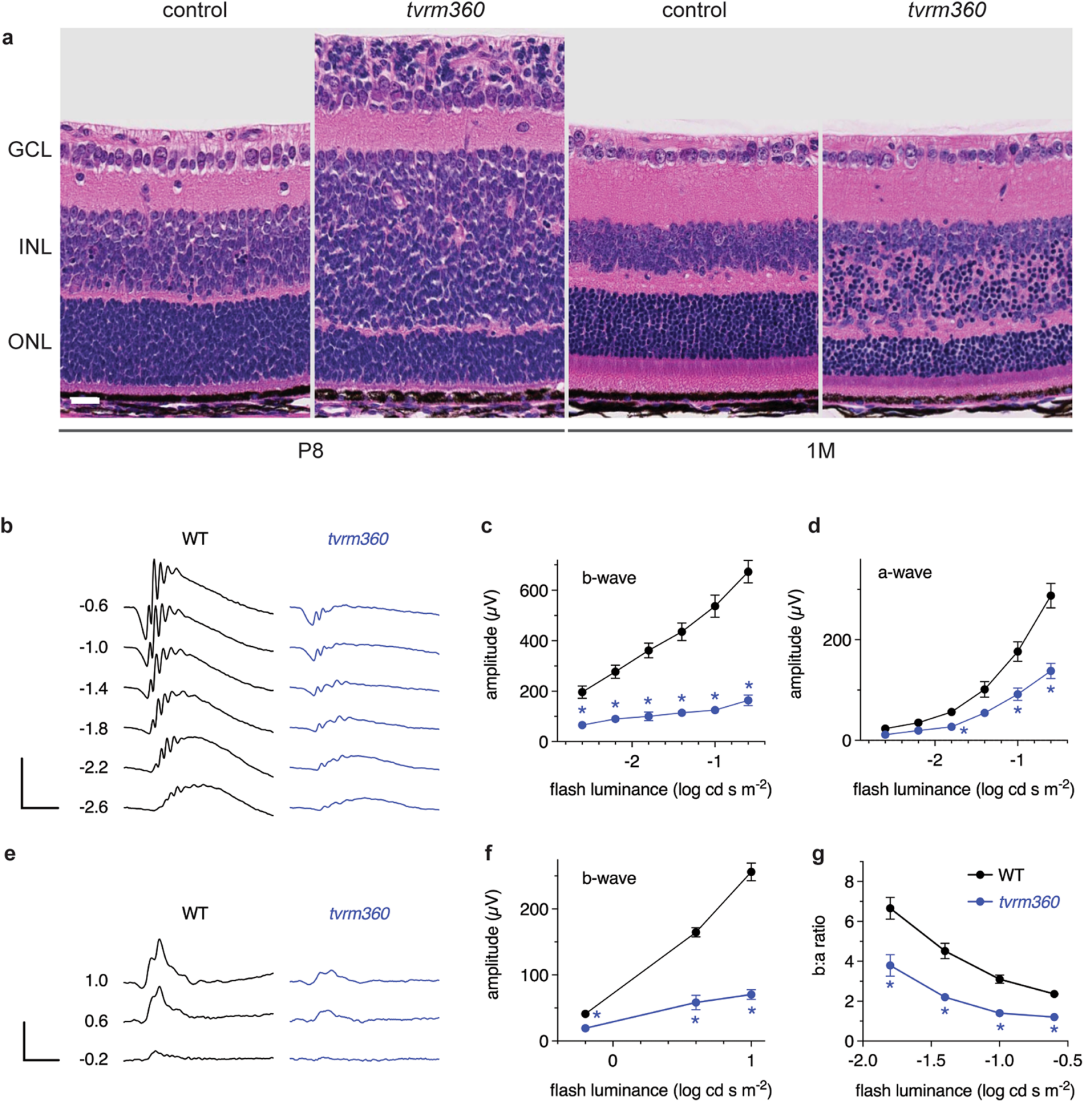
The laminar organization of the retina is perturbed in homozygous *Eml1*^*tvrm360*^ eyes. (**a**) Retinal sections of control eyes show dense hematoxylin-stained nuclei in the outer nuclear layer (ONL) at postnatal day (P) 8 and 1 M. In contrast, in *Eml1*^*tvrm360*^ mutant retinas dense hematoxylin-stained nuclei were also observed in the GCL at P8 and in the INL at P8 and 1 M. GCL, ganglion cell layer; ONL, outer nuclear layer; INL, inner nuclear layer. Scale bar: 20 µm. (**b–g**) Full field ERG responses recorded in *Eml1*^*tvrm360*^ (n = 5) and control littermates (n = 5) at one month of age. (b) Mean scotopic traces from a representative wild-type (WT, *black*) and homozygous *Eml1*^*tvrm360*^ littermate (*tvrm360*, blue) with increasing light stimulus intensities (values indicate flash illuminance in log cd s m-2). Scale bars: vertical, 500 µV; horizontal, 50 ms. Light dose response analysis showing mean ± SEM scotopic (**c**) b-wave and (**d**) a-wave amplitudes of WT (black circles) and *tvrm360* (blue circles) mice. (**e**) Representative photopic traces from the same mice as in (**b**). Scale bars: vertical, 200 µV; horizontal, 50 ms. (**f**) Light dose response analysis showing mean ± standard error photopic b-wave amplitudes in WT (black circles) and *tvrm360* (blue circles) mice. Asterisks indicate significant values (Pairwise t-test; rod b-wave P < 0.005, rod a-wave P < 0.05, cone b-wave P < 0.0005) between mutant and control. (**g**) Ratio of the scotopic b- and a-wave amplitudes (b:a ratio) from full field ERG recordings of *Eml1*^*tvrm360*^ and control littermates at 1 M of age. The b:a ratio is higher in controls than in mutant mice at all flash intensities evaluated (mean ± SEM), suggesting a defect in secondary neuronal signaling. Data were analyzed by a pairwise t-test. *P < 0.01.

While changes in the fundus were not observable by indirect ophthalmoscopy, histological examination of the retina revealed significant lamination defects. At P8, the developing outer plexiform layer (OPL) was aberrantly positioned in the midsection of the presumptive ONL of *Eml1*^*tvrm360*^ mice (Fig. 3a). Consequently, the outer neuroblastic layer appeared to be thinner in homozygous *Eml1*^*tvrm360*^ mutants compared to age-matched controls. In addition, in *Eml1*^*tvrm360*^ retinas, several dense hematoxylin-stained nuclei similar to those observed in the ONL were found ectopically positioned in the INL and the ganglion cell layer (GCL). We hypothesized that these were ectopic rod photoreceptor nuclei. In addition to the ectopic cells, the outer segments (OS), OPL and ONL were thinner, and the INL was thicker in mutant mice compared to controls at one month of age (Fig. 3a).

To examine the effects of the mutation on neural retinal function, we performed electroretinography on *Eml1*^*tvrm360*^ and control littermates at one month of age (Fig. 3). As shown in Fig. 3, scotopic (dark-adapted, 3b-d) and photopic (light-adapted, 3e,f) recordings show diminished rod and cone responses in *Eml1*^*tvrm360*^ retinas compared to WT littermates. The ERG b:a-wave ratio was significantly lower in *Eml1*^*tvrm360*^ retinas suggesting a defect in the secondary neuron responses (Fig. 3g).

### Ectopic photoreceptors and synapses are observed in *Eml1*^*tvrm360*^ mutants

**Figure 4:**
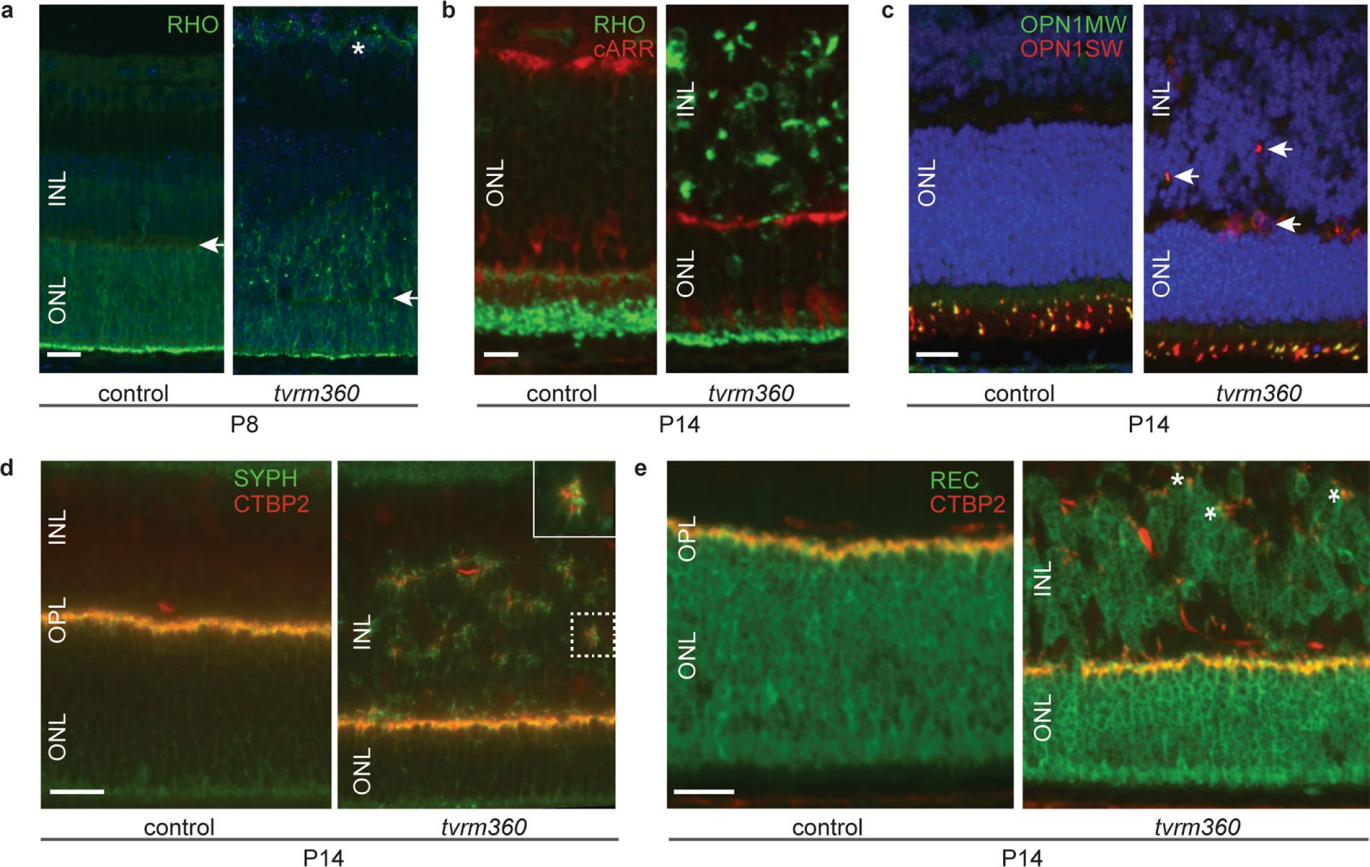
Ectopic cells in the INL of homozygous *Eml1*^*tvrm360*^ mice are rod and cone photoreceptors. (**a**–**c**) Retinal sections immunostained with photoreceptor-specific antibodies: rhodopsin (RHO), cone arrestin (cARR), blue opsin (OPN1SW) and green opsin (OPN1MW). Retinal staining at P8 and P14 shows mislocalization of visual pigments in the INL of *Eml1*^*tvrm360*^ mutants. (**a**) White arrow demarcates presumptive OPL while asterisks show mislocalized rod opsin in the GCL at P8. (**b**) Shortened cone processes in the ONL are revealed by cone arrestin staining which highlights the position of the cone pedicules in the presynaptic layer and cone cell bodies on the apical side of the ONL. (**c**) White arrows indicate mislocalized blue opsin pigment in the INL of *Eml1*^*tvrm360*^ mutant retinas. (**d**) Immunostaining with synaptic markers, SYPH and CTBP2 (ribeye) shows ectopic synapses in the INL of homozygous *Eml1*^*tvrm360*^ mice at P14. Closer view of boxed region in INL is shown in the top right inset (SYPH, synaptic vesicle; CTBP2, synaptic ribbon). (**e**) White asterisks indicate ectopic synapses (CTBP2) found adjacent to mislocalized photoreceptors (REC, recoverin) in mutant retinas. ONL, outer nuclear layer; INL, inner nuclear layer; OPL, outer plexiform layer. Scale bars: 20 µm (**a**,**c**–**e**); 10 µm (**b**).

To determine the nature of the mislocalized cells in the INL, a marker study was undertaken (Fig. 4). At P8, when outer segment development has initiated, rhodopsin staining was confined to the scleral half of the neuroblastic layer in controls, whereas, in homozygous *Eml1*^*tvrm360*^ mice, some rhodopsin was mislocalized to the outer neuroblastic layer and GCL (Fig. 4a). In normal retinal development, at P14, rhodopsin staining is found primarily in the OS of control mice. In contrast, there was significant rhodopsin staining in the OPL, INL, and GCL in mutants at this time point (Fig. 4b). In addition, staining of cone cell bodies and pedicules with cone arrestin in mutants showed that properly localized cone photoreceptors had shortened synaptic processes compared to age-matched controls. (Fig. 4b). Staining with cone pigment markers, OPN1SW and OPN1MW, revealed that blue and green opsin expression was restricted to the outer segment layer of control mice at P14, but ectopic blue cone pigments were occasionally observed in the INL of *Eml1*^*tvrm360*^ mutant retinas (Fig. 4c). Consistent with photoreceptor mislocalization to the INL, C-terminal binding protein 2 (CTBP2; ribeye) and synaptophysin (SYPH) staining, which normally marks photoreceptor synaptic ribbons and presynaptic vesicles in the OPL respectively, was found in the INL of *Eml1*^*tvrm360*^ mutant retinas (Fig. 4d). Co-immunostaining with anti-recoverin (a marker for photoreceptors) and CTBP2 showed ectopic synapses in the INL adjacent to the mislocalized photoreceptors (Fig. 4e).

**Figure 5:**
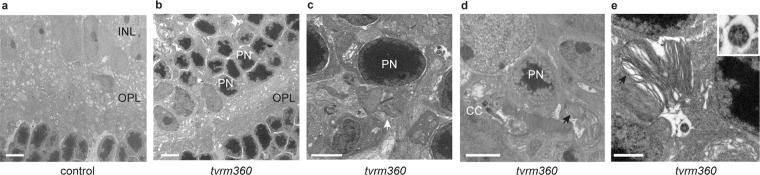
Ultrastructural abnormalities in homozygous *Eml1*^*tvrm360*^ mice at one month of age. Transmission electron micrographs (TEM) of the ONL-OPL-INL interface illustrate the abnormal positioning of rod photoreceptors (PN) in the INL of (**b**) *Eml1*^*tvrm360*^ mice retinas (n = 7) compared to (**a**) control littermates (n = 7). (**c**) High resolution micrograph showing a mislocalized synapse (white arrow) in the retinal INL of homozygous *Eml1*^*tvrm360*^. (**d**,**e**) The ectopic photoreceptors in the INL of *Eml1*^*tvrm360*^ mice develop normal “9 + 0” cilia (higher magnification shown in top right inset, panel **e**), but rudimentary and misshapen photoreceptor OS discs (black arrows). ONL, outer nuclear layer; INL, inner nuclear layer; CC, connecting cilium; PN, photoreceptor nucleus; OPL, outer plexiform layer. Scale bars: 5 μm (**a**,**b**); 2 μm (**c**–**e**).

Ultrastructural examination of retinas confirmed the presence of ectopic photoreceptors and synapses in the INL of *Eml1*^*tvrm360*^ mice (Fig. 5). Although synapses were observed between ectopic PR and secondary neurons (Fig. 5c), *Eml1*^*tvrm360*^ retinas had disproportionately lower ERG b:a-wave ratios (Fig. 3g). Interestingly, the mislocalized photoreceptors formed cilia with misshapen outer segments (Fig. 5d,e and Fig. S2a). The ciliary structure of both ectopic photoreceptors and photoreceptors positioned within the ONL appeared normal, showing the typical 9 + 0 microtubule arrangement and the presence of microtubule-associated vesicles (Fig. 5e right panel, inset). The outer segments of photoreceptors localized in the ONL were normal in length and arrangement in *Eml1*^*tvrm360*^ mice (Fig. S2b), however, the outer segments of ectopic photoreceptors were short and misshapen.

### Müller glia and inner retinal neurons are affected by the *Eml1* disruption

**Figure 6:**
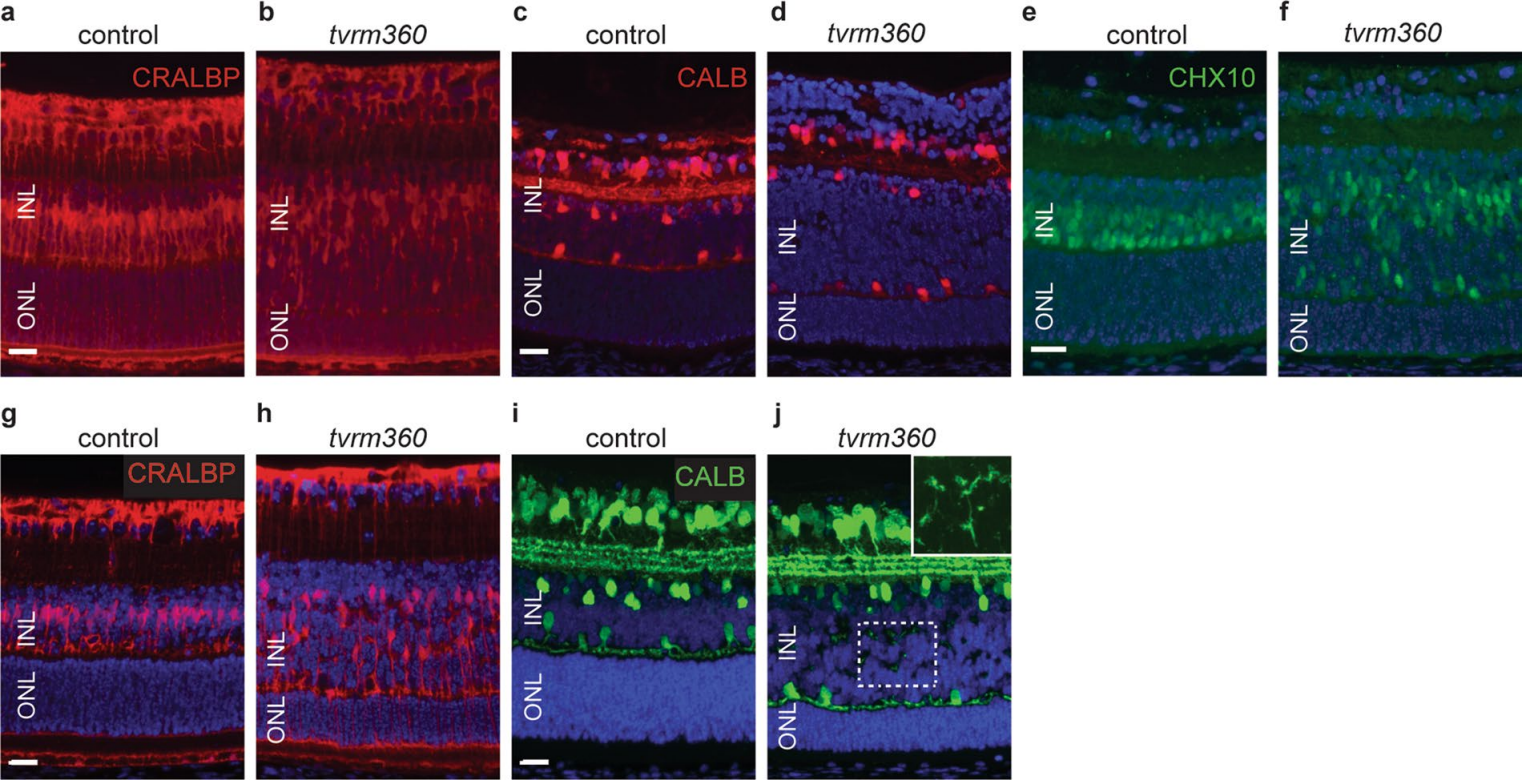
Disruption of *Eml1* affects the lamination of the inner retina. Immunostaining with anti-CRALBP at P8 and P14 shows a uniform Müller cell somata distribution in the midzone of the INL in control retinas (**a**,**g**), whereas, Müller cell bodies are disorganized and found throughout the INL of *Eml1*^*tvrm360*^ retinas (**b**,**h**). *Eml1*^*tvrm360*^ and control littermate retinas were stained with anti- calbindin and CHX10 (VSX2) to mark horizontal and bipolar cells, respectively (**c**–**j**). At P8, bipolar cells were found abnormally scattered throughout the INL in *Eml1*^*tvrm360*^ mice (**f**) compared to control littermates (**e**), however horizontal cell bodies appeared to be positioned correctly in *Eml1*^*tvrm360*^ retinas (**d**). (**j**) At P14, vertically positioned neurites were found scattered through the midzone of the INL in *Eml1*^*tvrm360*^ mutants. At the upper right corner panel, a magnified view of INL calbindin staining (dotted box) is shown without DAPI fluorescence. *Top row*, P8; *bottom row*, P14. ONL, outer nuclear layer; INL, inner nuclear layer. Scale bars: 20 μm.

To test whether other cell types were mispositioned in the *tvrm360* retina, we examined the distribution of Müller glia and inner retinal neurons by retinal marker analysis (Fig. 6). The distribution of cellular retinaldehyde binding protein (RLBP1, also known as CRALBP), a marker for Müller glia in the developing retina^23–25^ was examined. At P8 (Fig. 6a,b) and P14 (Fig. 6g,h), Müller cell bodies were uniformly aligned in the mid-section of the INL in control littermate retinas, whereas Müller cell bodies were found scattered throughout the INL in *Eml1*^*tvrm360*^ retinas.

During normal murine development of horizontal cells, vertical dendrites are typically replaced by lateralized dendrites at P14 as a result of maturation^26^. We used anti-calbindin and anti-CHX10 antibodies to detect horizontal and bipolar cells, respectively. Although, horizontal cell bodies in the *Eml1*^*tvrm360*^ mutant were positioned similarly to littermate controls at P8 (Fig. 6c,d), rudimentary vertical neurites of horizontal cells in the midzone of the INL were still evident in *Eml1*^*tvrm360*^ retinas at P14 (Fig. 6i,j). In addition, lamination defects of bipolar cells were observed in developing *Eml1*^*tvrm360*^ retinas as early as P8 (Fig. 6f).

### Ectopic mitotic progenitor cells are found in the neuroblastic layer (NBL) of *Eml1*^*tvrm360*^ mice

**Figure 7:**
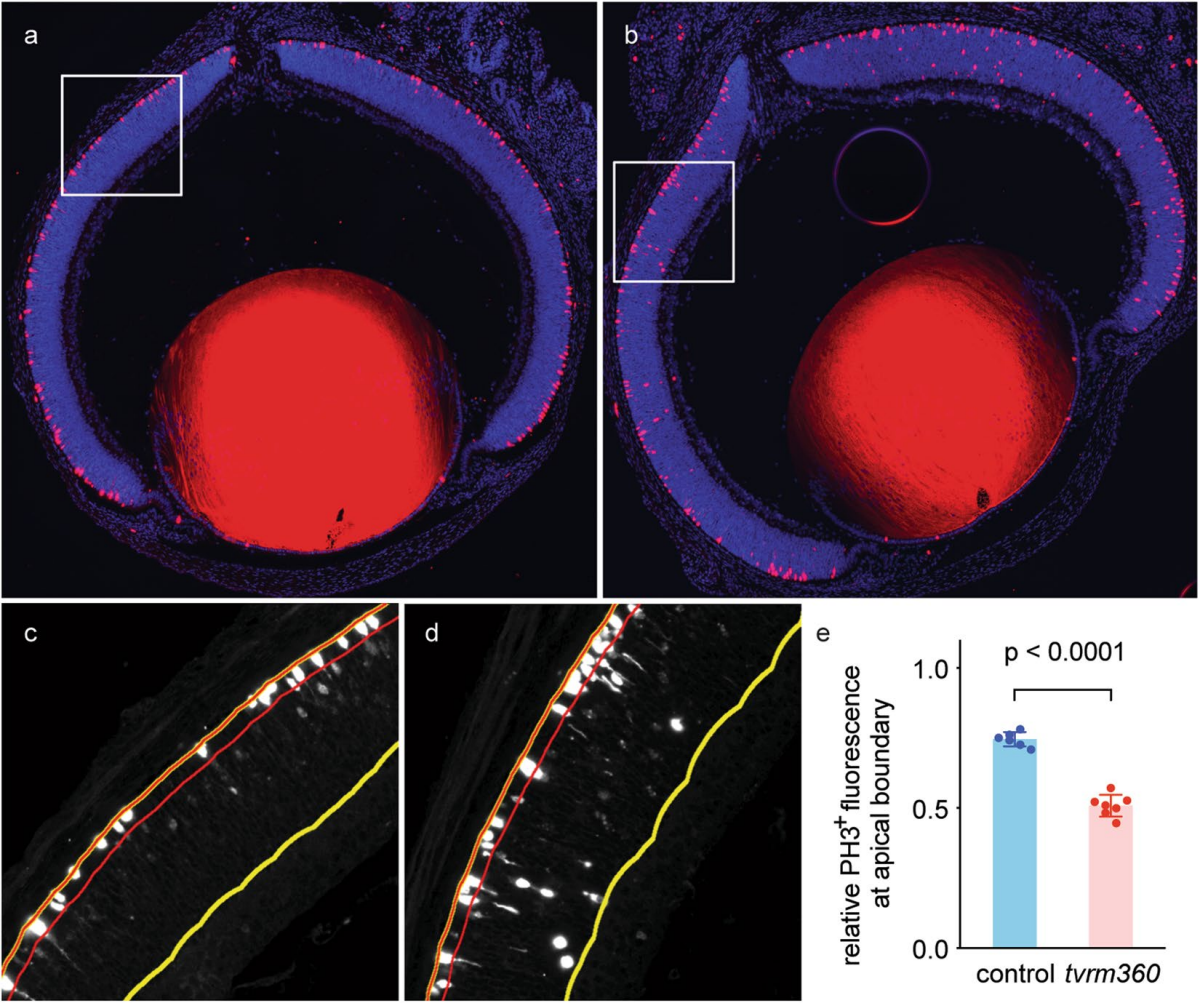
Displaced mitotic cells in developing retinas of homozygous *Eml1*^*tvrm360*^ mice. (**a**,**b**) Immunolocalization of mitotic cells in ocular sections with anti-PH3 antibody staining (*red*) in the NBL of *Eml1*^*tvrm360*^ mice (**b**) and control littermates (**a**) at birth (P0). Nuclei were counterstained with DAPI (*blue*). Boxed regions measure 300 µm per side. (**c**,**d**) The PH3 channel of the boxed regions in a and b, respectively, is presented in grayscale to visualize weakly stained cells. The selected boundaries of the apical NBL (*red*) and total NBL (*yellow*) are shown. (**e**) Quantitation of displaced mitotic cells from the apical boundary of the NBL in *Eml1*^*tvrm360*^ (n = 7) and control littermates (n = 6), including one heterozygous and five wild-type mice. Total PH3 + fluorescence at the apical NBL was divided by that of the entire NBL.

Recently, Bizzotto *et al*. demonstrated that lamination defects in *Eml1*^*heco*^ mutant neocortex was a result of improperly positioned mitotic spindles^27^. To determine whether there were any abnormalities in the distribution of progenitor cells undergoing cell division in the retina, retinal sections of homozygous *Eml1*^*tvrm360*^ and control littermates were stained with PH3 antibody, a marker for mitosis. At birth (P0), PH3-positive progenitor cells were found primarily at the apical boundary of the neuroepithelium of wild-type and heterozygous control mice (Fig. 7a,c). Although apical PH3-positive cells were observed in *Eml1*^*tvrm360*^ retinas, a substantial number appeared in other regions of the NBL (Fig. 7b,d). Counting individual PH3-positive cells was difficult due to the variability of PH3 fluorescence intensity among cells (Fig. 7c,d), a likely consequence of the known variation in PH3 levels with mitotic stage^28,29^. Therefore, to quantify the altered distribution of mitotic cells, we measured the ratio of PH3 fluorescence within the apical region (Fig. 7c,d, *red lines*) to that observed within the total NBL Fig. 7c,d, *yellow lines*). A statistically significant decrease in this ratio was observed in homozygous *Eml1*^*tvrm360*^ mice (Fig. 7e), indicating a lamination defect of apical progenitor cells.

## Discussion

Proper lamination of tissues of the central nervous system is essential for normal development. In the neocortex and retina, lamination occurs through a process whereby neuroepithelial cells undergo a coordinated series of events involving cell division, cell-fate determination, migration, and terminal differentiation^30^. Apical precursors move through the developing neuroepithelia via a process known as interkinetic nuclear migration to form highly specialized cellular layers^4^. Proper apico-basal polarity and mitotic cell positioning are essential for normal migration and morphogenesis of neuroepithelial progenitors^5^. During this process, the placement of proliferating nuclei along the ventricular zone is critical for cell cycle fate, *i.e*. control of whether precursors remain in a proliferative (mitotic) state or a neurogenic (differentiation) state^2,31^. Disruption at any stage during neuroepithelial development may result in lamination defects of the neocortex and/or retina.

In this study, we report a novel finding of retinal lamination defects found in association with a disruption in *Eml1*, a member of a highly conserved echinoderm microtubule associated (EMAP) gene family^32^. Members of the EMAP family, including EML1 are known to play key roles in loading cargo onto microtubules, such as components of the kinetochore machinery onto the spindle apparatus during cellular proliferation^33–35^. Specifically, the EML1 protein has a unique hydrophobic EMAP-like (HELP) domain known to interact with microtubules, and several imperfect WD40 repeats which fold into propeller-like structures^36,37^. These structures are thought to function in vesicular transport, signal transduction, cytoskeletal organization, and transcriptional and cell cycle regulation. Recent studies have suggested roles for EML1 in primary cilia formation, microtubular cytoskeletal assembly and organization, regulation of mitotic spindle length/positioning and of neuronal progenitor proliferation^15,27,38^. Our studies show *Eml1* is expressed early in mouse development in a variety of tissues including the eye. Quantitative PCR confirmed the presence of both the long and short forms of *Eml1* in the retina. While the expression of the shorter form(s) were significantly decreased in the *Eml1*^*tvrm360*^ retinas compared to B6J controls at two weeks of age, it is intriguing that the longer form does not appear to undergo nonsense mediated decay. Since the primers are specific to the 5′ end of *Eml1* (unique to the long and short forms), it remains possible that there may be retinal-specific splice variants that exclude the mutation in exon 18. This idea is further supported by findings that during development, an abundance of alternative splice isoforms are selectively expressed in the retina^39–41^. To better understand the molecular significance of these expressed transcripts in *Eml1*^*tvrm360*^ during retinal development, further characterization of *Eml1* full length transcripts in retinal tissue is warranted. In addition, the generation of antibodies that are specific for the long and shorter isoforms, will help to decipher whether these variants are transcribed into a stable protein. It is noteworthy that although commercial antibodies failed to detect EML1 in murine retinal tissue by immunohistochemistry, archived data of the mouse proteome, lists EML1 as a component of the photoreceptor sensory cilium complex^42^.

A role for EML1 in retinal photoreceptors has been demonstrated in non-mammalian species. The striped bass ortholog of EML1, CNG-modulin, interacts with the cGMP-gated channel and regulates calcium-dependent cGMP sensitivity in cone photoreceptors^43^. In both striped bass and zebrafish, CNG-modulin has been shown to express in cone photoreceptors^43,44^. Interestingly, continuous illumination studies in zebrafish morpholino-induced knockdown of *Eml1* demonstrate extreme light sensitivity in dark adapted cones^44^, suggesting an important role of protein in ensuring proper retinal function. However, in contrast to our observations in *Eml1*^*tvrm360*^ mice, *Eml1* zebrafish morphants do not appear to display retinal lamination defects^44^. The phenotypic variability may be attributed by the differences in CNG-modulin/EML1 expression. Mice have three protein coding transcripts of EML1^21^. Because the long and short forms of murine *Eml1* are differentially expressed, it is likely that they may have different functions. While the longer form shows ubiquitous expression, the shorter forms have restricted spatial and temporal expression in the eye and brain; their tissue-specific roles remain to be elucidated.

The abnormalities we observed in the retina of *tvrm360* mice have not been reported in other murine models with *Eml1* mutations^17^. However, the retinal lamination defects in our mice bear phenotypic similarities to the cerebral lamination defects in the *Eml1* mutants previously described^17^. Kielar *et al*. have shown that in *Eml1*^*heco*^ mice, heterotopia results from cortical neuron loss due to improper spindle orientation and aberrant positioning of their radial glia progenitors^15^. Defects in retinal architecture were observed as early as P0 in *Eml1*^*tvrm360*^ retinas. Analysis of mitotic nuclei in *Eml1*^*tvrm360*^ retinas revealed defects in the positioning of proliferative progenitor cells resulting in defective apico-basal polarity and interkinetic nuclear migration. During retinal development, the apical end of the neuroepithelium consists mainly of mitotic progenitor cell nuclei adjacent to the adherens junctions and centrosomes. The apical positioning of centrosomes and apico-basal polarity are crucial for proper nuclear migration^5^. Although the exact mechanisms driving apical centrosome positioning are not certain, the centrosomes are thought to be anchored to the apical surface by a primary cilium^45^. Disruption of proteins leading to defects in spindle orientation have ectopically positioned centrosomes and perturbed nuclear migration^46,47^. At birth, mitotic retinal cells of homozygous *Eml1*^*tvrm360*^ were displaced within the neuroblastic layer. The ectopic localization of the centrosomes observed during cellular proliferation suggests that EML1 may be crucial in establishing apico-basal polarity and may likely play a role during the cell cycle, perhaps by tethering centrioles to the microtubules at the apical surface. Further, these findings indicate that EML1 contributes to the proper positioning of progenitor cells during early postnatal retinal development and raises the possibility that the *Eml1*^*tvrm360*^ mutation alters progenitor cell fate by disrupting the postulated role of EML1 in mitotic spindle positioning.

During postnatal development, some ectopic photoreceptors were found in the INL and GCL of *Eml1*^*tvrm360*^ retinas. Normally, a small population of ectopic photoreceptors are found in the GCL and INL during the first postnatal week, likely a result of migration errors^48^. However, these misplaced cells in the GCL are typically eliminated by P7 while those in the INL are removed by P14, a time at which mouse photoreceptors are reaching maturation. In contrast, ectopic photoreceptors remained in the INL and GCL of *tvrm360* mutants at two and four weeks of age.

Most recently, a role for EML1 in cilia formation has been elucidated by Uzquiano *et al*.^38^. In their study, structural defects were observed in primary cilia from apical radial glial cells of *Eml1*^*heco*^ mice and from fibroblasts of *EML1*-heterotopia patients. The group further proposed that the ciliary malformations may be a consequence of impaired protein trafficking from the Golgi apparatus. In our model, the photoreceptor cilia formed properly with a normal “9 + 0” microtubular arrangement. The normally positioned photoreceptor cells in the outer nuclear layer of *Eml1*^*tvrm360*^ mutants were juxtaposed with the retinal pigmented epithelium and produced normal connecting cilia and outer segments. However, it is interesting to note that while the ectopic photoreceptors of *Eml1*^*tvrm360*^ mutants were able to form normal cilia, the outer segment discs failed to develop properly. Recent studies have demonstrated the importance of RPE apico-basal polarity in photoreceptor differentiation and homeostasis^49^. In our model, ectopic outer segment discs in the inner nuclear layer do not lie in close proximity to the retinal pigmented epithelium. Conceivably, the failure of outer segment biogenesis may be due to improper RPE signaling required for the maintenance of these ectopic photoreceptor discs. While it remains to be determined whether there is aberrant Golgi trafficking in *Eml1*^*tvrm360*^ retinas, we cannot exclude the possibility that failed delivery of critical proteins involved in adherens junction formation of the external limiting membrane may also contribute to the ectopic positioning of photoreceptors.

In conclusion, our finding of ectopic mitotic progenitors in the *Eml1*^*tvrm360*^ model during postnatal retinal development extends previous observations that EML1 may function in microtubule-related processes that are essential for neuronal lamination, such as the positioning of retinal progenitor cells during interkinetic nuclear migration and control of progenitor developmental fate by mitotic spindle orientation^50,51^. Unraveling the molecular pathways that lead to proper apico-basal polarity and pseudostratification of the developing neuroepithelium will give us a better understanding of the pathophysiological basis of retinal lamination defects and the resulting effects on vision.

## Methods

### Ethics statement

Procedures used in the experiments were approved by the JAX Institutional Animal Care and Use Committee (IACUC; protocol number 99089) and were conducted in accordance with the guidelines established by the National Institute of Health (Bethesda, Maryland) and the Association for Research in Vision and Ophthalmology for the Use of Animals in Ophthalmic and Vision Research.

### Mouse husbandry and origin

Mice were bred and housed in the Research Animal Facility at The Jackson Laboratory and monitored regularly to maintain a pathogen free environment. Mice were provided a NIH 6% fat chow diet and acidified water *ad libitum*, and housed in a vivarium with a 12:12 hour dark:light cycle. The *tvrm360* mutant, which develops hydrocephalus and an inner nuclear layer defect, was identified in a N-ethyl-N-Nitrosurea (ENU) mutagenesis screen of mice generated in the TVRM program at The Jackson Laboratory^18^. Originally, the *tvrm360* mutant, was identified in a colony of mice that was homozygous for a *Rp1*^*tvrm64*^ (*tvrm64*) mutation^18^. A mapping cross involving C57BL/6J-*Rp1*^*tvrm64*^ and DBA/2J (D2) mice was used in both the initial and the subsequent high resolution linkage analysis. To isolate the *tvrm360* mutation from the *Rp1*^*tvrm64*^ allele, we selected for mice that did not carry the *tvrm64* allele, as we backcrossed onto the C57BL/6J (B6) background.

### Chromosomal localization and fine structure mapping

Genomic DNA was isolated from tail tips, as previously described^52^. DNAs of 11 affected F2 offspring were pooled and subjected to a genome-wide scan in which ~80 robust simple sequence length polymorphic (SSLP) markers known to differ between B6 and D2 were used. Once a map position was identified on Chr 12, samples were tested individually to confirm the linkage. In the subsequent high-resolution intercross involving 174 F2 mice, informative recombinant mice were examined histologically for lamination defects, and selected uninformative recombinants were progeny tested by crossing them to B6-*tvrm360*/+ mice to determine if they carried the disease gene. A minimum of 20 offspring from each progeny test were genotyped and phenotyped. For PCR amplification, 25 ng DNA was used in a 10 μl volume containing 50 mM KCl, 10 mM Tris-Cl, pH 8.3, 2.5 mM MgCl_2_, 0.2 mM oligonucleotides, 200 µM dNTP, and 0.02U AmpliTaq DNA polymerase. The reactions that were initially denatured for 2 min at 95 °C were subjected to 49 cycles of 20 s at 94 °C, 20 s at 50 °C, 30 s at 72 °C and a 7 min extension at 72 °C. PCR products were separated by electrophoresis on a 4% MetaPhor agarose gel (Lonza) and visualized under UV illumination after staining with ethidium bromide. Mutant and wild-type mRNA was obtained from whole-eye tissues using TRIzol reagent, according to the manufacturer’s directions (Life Technologies), and template cDNA was generated with oligo(dT) primers. We designed exon-spanning primer pairs and used them to amplify the coding region of *Eml1*. RT-PCR was performed in an MJ Research PTC-200 or Eppendorf Mastercycler (Thermo Fisher Scientific) using the following program: initial denaturation at 94 °C for 2 min; then 35 cycles of 94 °C for 30 s, 56 °C for 30 s, and 68 °C for 2 min. The resulting product was electrophoresed on a 1% agarose gel and isolated using a NucleoSpin Extraction kit (Clontech). The purified DNA was sequenced using Big Dye Terminator Cycle Sequencing Chemistry on a PE Applied Biosystems 3600.

### Allele specific PCR

The following genotyping protocol was used to detect the *Eml1* mutation. Oligonucleotides (emlgR3, GGTAAGTTTCTCTTGCCTTTCTGA; emlASF3, CCCATGACAACTGCATCTACATATGA; and emlASF4, GCAATCGGCTGGCATGACAACTGCATCTACATGTAT) were designed to amplify the wildtype (178 bp) and mutant (168 bp) alleles of *Eml1* by allele specific PCR. Cycling conditions were as follows: Initial denaturation at 94 °C for 2 min, 50 cycles of 94 °C for 20 sec, 55 °C for 30 sec, and 72 °C for 30 sec followed by a final extension of 72 °C for 5 min. Amplified products were resolved in a 4% MetaPhor agarose gel (Lonza), and visualized by ethidium bromide staining under UV illumination.

### RNA isolation and RT-PCR analysis

#### RNA isolation

Total RNA was isolated from postnatal day 14 (P14) eyes of C57BL/6J (n = 5) and *Eml1*^*tvrm360*^ mice (n = 5) using TRIzol reagent (Life Technologies), followed by treatment with RNase-free DNaseI (Ambion). RNA quantity and quality was assessed using a Nanodrop spectrophotometer (Thermo Fisher Scientific) and a 2100 Bioanalyzer (Agilent Technologies), respectively. cDNA was made using the Retroscript kit (Ambion) according to manufacturer’s protocol.

#### RT-PCR

The long and short forms differ by the locations of their initiation ATG codons.

For transcript-specific PCR of murine *Eml1*, primers were designed such that the forward primer was specific to the unique first exon of each form and a common reverse primer overlapped exons 2–3: short forms, (2F6), AGCCTGGATGGGAAGAGG), long form, (LF1, AGCTATAGCAGCCTGTACGACAC) and common reverse primer (CR1, CTCGCTTTGGTAGGTCCTTTCC). For tissue-specific expression analysis, we used a Mouse MTC Panel (cat 636745, Clontech) as template for RT-PCR. In addition, we generated cDNA from RNA isolated from whole eyes and retinas of C57BL/6J and *Eml1*^*tvrm360*^ mice at postnatal day 14. To isolate the neural retina, eyes were removed and immediately placed in ice cold 1X PBS containing 10 mM Ribonucleoside Vanadyl Complex (S1402S, New England Biolabs). The optic globe was circumferentially cut below the limbus and the anterior chamber and lens were removed. The posterior eyecup was incubated in 220 U/ml hyaluronidase (H3506, Sigma) and 10 mM Ribonucleoside Vanadyl Complex in 1X PBS on a rotator for 10 min at 37 °C. Following incubation, the neural retina was separated from the RPE/choroid/sclera complex and immediately flash frozen in liquid nitrogen.

PCR was carried out using cDNA in a 20 μl PCR reaction containing 1xPCR buffer (10 mM Tris-HCl pH 8.3, 50 mM KCl), 250 μM of dATP, dCTP, dGTP, and dTTP, 0.2 μM of the forward and the reverse primer, 1.5 mM MgCl_2_, and 0.6 U Taq polymerase. The following PCR program was used: 94 °C for 90 sec, followed by 35 cycles of 94 °C for 30 sec, 55 °C for 45 sec, and 72 °C for 45 sec with a final extension of 72 °C for 2 minutes. For quality control, template cDNA was amplified with primers specific to 18S RNA (18S-F, AGGAGTGGGCCTGCGGCTTA and 18S-R, AACGGCCATGCACCACCACC). Amplification products were separated by gel electrophoresis and visualized by ethidium bromide staining under UV illumination. Images were digitally inverted in the acquisition software of the GeneFlash gel documentation system (Syngene) or in Fiji^53^. Brightness and contrast were adjusted in Fiji. Full length RT-PCR gels are shown in Supplementary Information.

For quantitative RT-PCR analysis of *Eml1*^*tvrm360*^ and control whole eyes, qRT–PCR was performed with the iTaq Universal SYBR Green SuperMix (Bio-Rad) and gene-specific primers positioned at the 3′end of *Eml1* to detect all 3 isoforms (Eml1-ex19F, TGGGTTCCGTCTGCCTGTAA and Eml1-ex21R, GGTGCACTTTGCCGAAGTCA) using the CFX96 Real-Time PCR Detection System (Bio-Rad). The comparative CT method (ΔΔC_T_) was used to calculate a relative fold change in gene transcripts. Quantification was performed using 2^*−ΔΔCT*^ with *Actb* as an internal control calibrator. Accurate amplification of the target genes was verified by melting curve analyses.

### Histological and immunohistochemical analysis of ocular tissue

The protocols for histological and immunohistochemical assays were previously described^54^. Briefly, mice were sacrificed by carbon dioxide asphyxiation. Enucleated eyes were placed in cold acetic acid/methanol or 4% paraformaldehyde solution in PBS overnight followed by paraffin embedding using standard procedures. Tissues were cut into 4 µm sections, stained with hematoxylin and eosin (H & E) and examined by light microscopy. Stained tissue images were gathered using a NanoZoomer slide scanner (Hamamatsu).

For immunohistochemistry, deparaffinized sections were blocked with 1:50 normal goat serum, 0.3% Triton X-100 in phosphate buffered saline (PBS) followed by incubation with the following antibodies: mouse anti-rhodopsin (Leinco Technologies 1:500 or Thermoscientific, 1:1), goat anti-CHX10 (Santa Cruz 1:100), goat anti-S opsin (Santa Cruz, 1:200), rabbit anti-M opsin (Chemicon, 1:200), mouse anti-PKC α (Abcam, 1:100), rabbit anti-phospho histone H3 (Abcam 1:200), rabbit anti-synaptophysin (Epitomics, 1:100), rabbit anti-CRALBP (a generous gift from Dr. Saari, 1:50), mouse anti-CTBP/ribeye (BD Biosciences, 1:500), and rabbit anti-calbindin (Abcam, 1:300). Specific proteins were fluorescently labeled by Cy3-conjugated (Jax ImmunoResearch) or Alexa Fluor 488 conjugated (Thermo Fisher) secondary antibodies. Tissue sections were stained with DAPI nuclear stain, mounted in Vectashield (Vector Lab) and visualized by fluorescence microscopy.

To quantify the distribution of PH3-positive cells, whole retinal sections were imaged at 10x on a Zeiss Axio Observer.Z1 by acquiring six tiles as image stacks of 15 z-steps with a step size of 2 µm. All images were acquired with identical fluorescence settings optimized for each channel. Using a custom Fiji/ImageJ macro, both channels of the resulting images were projected using a 3D enhanced depth-of-focus projection algorithm^55^, the PH3 channel was stitched^56^, and the stitching transforms applied to the DAPI channel. To determine PH3-positive cell distribution, a second macro was used to define a region of interest (ROI) in the DAPI channel corresponding to the total neuroblastic layer (NBL) by manual selection with the Wand tool. This ROI was edited to create a second 15-pixel-wide ROI that encompassed approximately two cell bodies at the apical NBL. The PH3 channel was processed with Subtract Background (20 pixels radius) and the ratio of integrated fluorescence intensity within the apical ROI to that within the total NBL ROI was determined. A Student’s t-test was used to assess statistical significance.

### Transmission electron microscopy (TEM)

For TEM analysis, animals were perfused trans-cardially with PBS followed by a 2.5% gluteraldehyde/2% paraformaldehyde fixative solution in phosphate buffer. The eyes for ultrastructural analyses were fixed in an ice cold fixative solution for 3 h. The anterior segment was removed and the posterior segment cut into 1 × 2 mm blocks. Additional fixation with 0.25% glutaraldehyde/0.2% paraformaldehyde fixative was performed for 2–8 h followed by post-fixation with 1% osmium tetroxide. The dehydrated blocks were embedded in plastic. Tissue sections were cut and stained with uranyl acetate and lead citrate and examined with a JEM-1230 transmission electron microscope (JEOL, Ltd).

### Electroretinography (ERG)

One month old homozygous *Eml1*^*tvrm360*^ mice and wildtype (WT) control littermates were examined by full field ERG recordings as previously described^13,57^. Briefly, after 2 hours of dark adaptation, mice were anesthetized with ketamine (80 mg/kg) and xylazine (16 mg/kg) diluted in normal saline, and pupils were dilated with 1% atropine (Alcon). To maintain body temperature, mice were placed on a platform maintained at 37 °C throughout each recording session. A gold recording electrode was placed on the corneal surface and referenced to a thin wire placed on the cheek, and a thin needle wire placed in the tail served as the ground electrode. Signals were differentially amplified, averaged, and stored using an Espion E3 Electroretinography system (Diagnosys, LLC). Rod-mediated ERG responses were recorded in mice dark-adapted for a minimum of 2 hours. Flash stimuli were presented in a Ganzfeld ColorDome (Diagnosys LLC) and ranged in luminance from −2.6 to −0.6 log cd s/m^2^. Five flashes were averaged at each stimulus intensity. After an 8-min period of light adaptation^58^, cone-mediated ERG responses were recorded with flashes of white light (6500 K) superimposed on the rod saturating background. Flash luminance ranged from −0.2 to 1 log cd s/m^2^ and responses to 25 records were averaged at each intensity level. ERG data was analyzed using a pairwise t-test with correction for multiple tests (Holm-Sidak method).

## Supplementary information

Supplementary Information.

## Data Availability

The datasets generated and/or analyzed for the current study are available from the corresponding author.
