# Supplementary material for: Disruption in murine Eml1 perturbs retinal lamination during early development
Source: Sci Rep. 2020 Mar 27;10:5647. doi: 10.1038/s41598-020-62373-5 (PMC7101416; doi:10.1038/s41598-020-62373-5)
Supplement: Supplementary file 1 — Supplementary Information. [file 41598_2020_62373_MOESM1_ESM.pdf]

Disruption in murine *Eml1* perturbs retinal lamination during early development

Collin GB[1,2]*, Won J[1,2], Krebs MP[1], Hicks, WJ[1], Charette, JR[1], Naggert JK[1], and Nishina PM[1]*

[1]The Jackson Laboratory, Bar Harbor, ME 04609

[2]Authors contributed equally to this work described.

*Corresponding authors

Supplementary figures

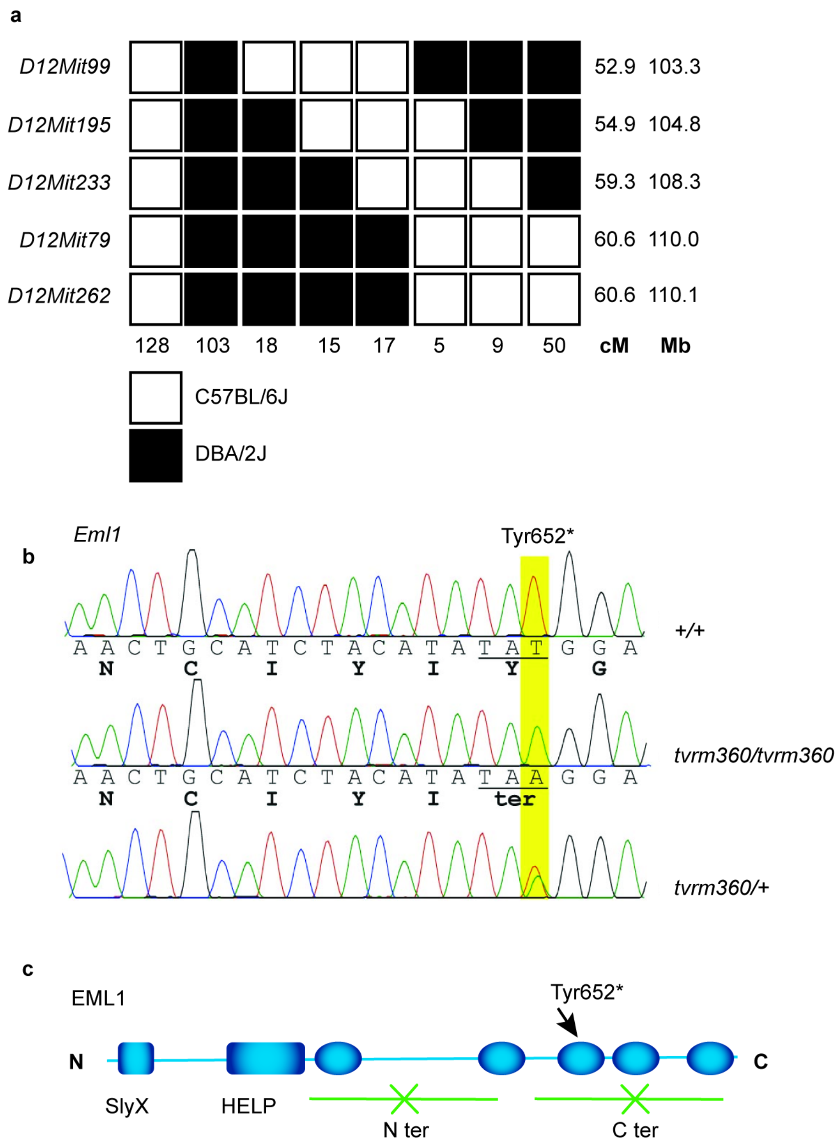

Fig S1. Chromosomal localization of the *tvrm360* allele*.* (**a**) Recombinational mapping revealed a minimal region on mouse chromosome 12 between *D12Mit99* and *D12Mit262* that harbored the mutation. Subsequently, fine structure mapping identified a critical interval containing fourteen protein coding

transcripts. (**b**) Direct sequencing of the candidate gene, *Eml1*, in wildtype, heterozygous carriers and mutants revealed a T/A point mutation in *tvrm360* resulting in an early termination codon (Tyr652*). (**c**) The mutation lies within the C-terminal propeller (C ter) of the TAPE domain[1].

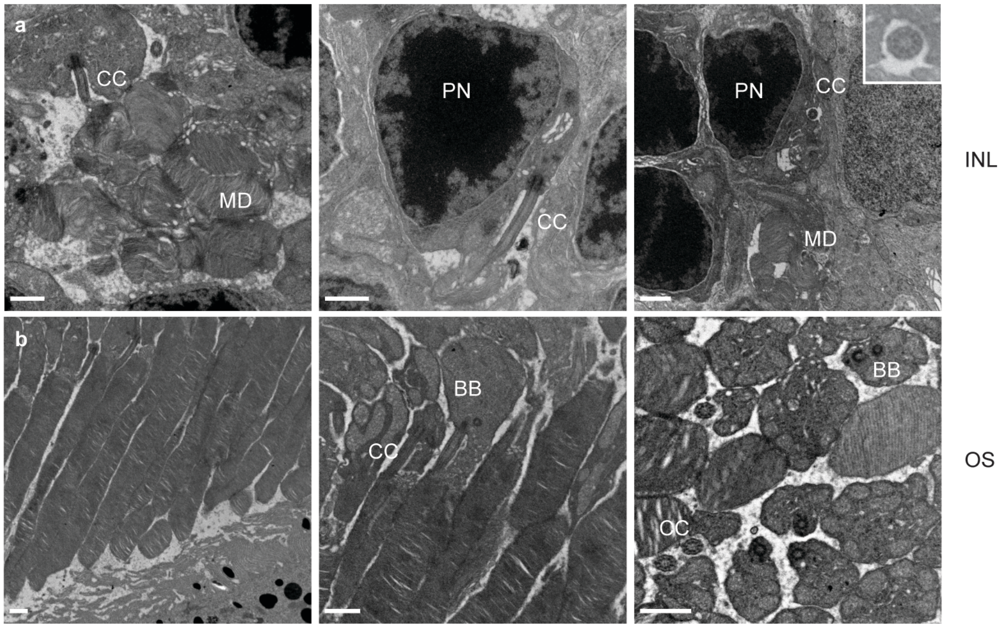

Fig S2. Transmission electron micrographs of *Eml1*^{tvrm360} retinas. (**a**) Ectopic photoreceptor nuclei (PN), connecting cilia (CC) and rudimentary membrane discs (MD) are observed in the INL at one month of age. High resolution images show fusion of basal bodies (BB) to membrane and ciliary axoneme extension into ciliary vesicles. Inset: Cross sectional view showing typical “9+0” microtubular arrangement. (**b**) *Eml1*^{tvrm360} photoreceptors that reside in the ONL form properly arranged outer segment discs and connecting cilia (CC). Scale bars: 1 μm.

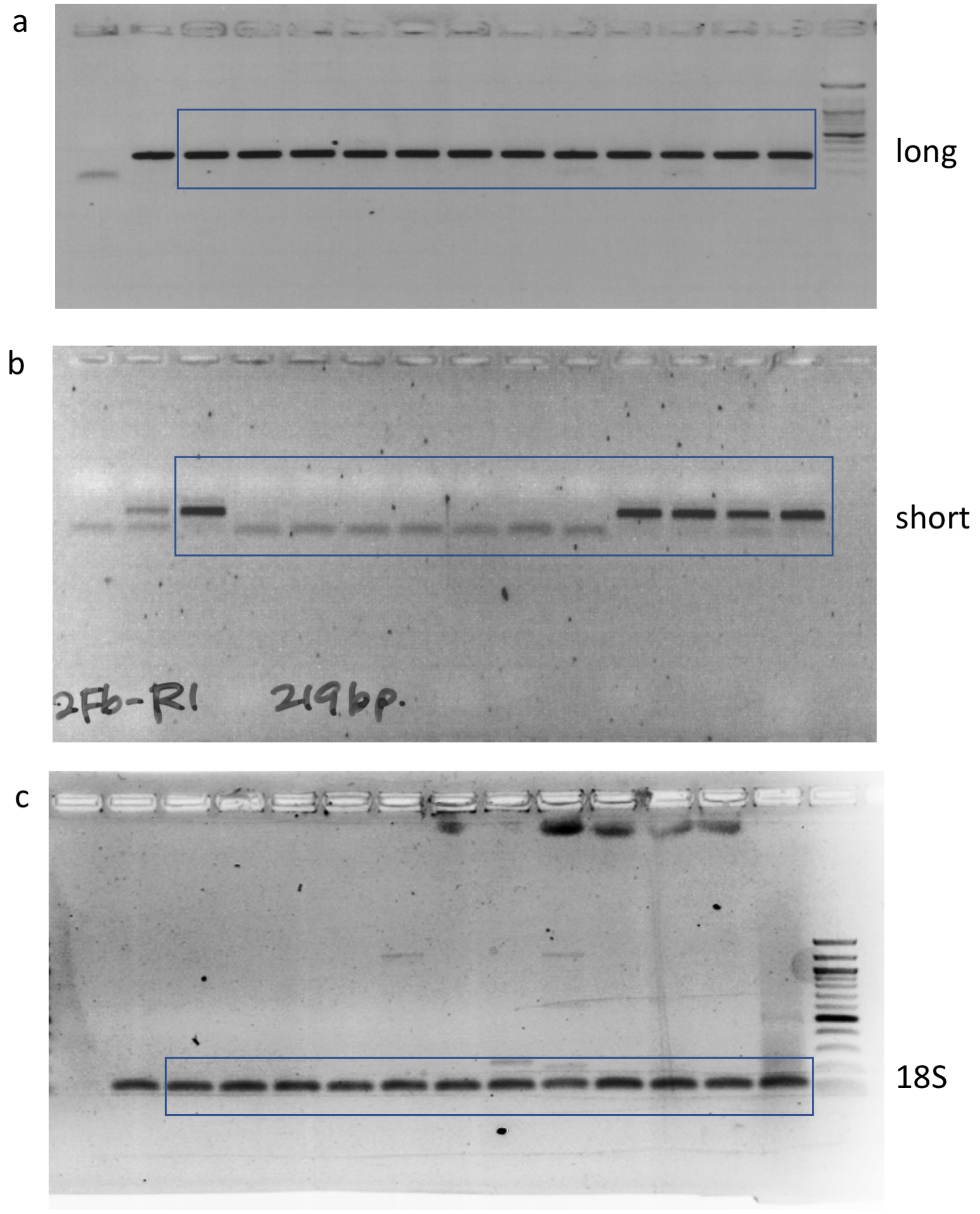

Fig S3. Uncropped gel images from Figure 1a.

Supplementary Table S1

**Gene**	**GRCm38 coordinates (bp)[2]**	**Genomic position (cM)**	**Murine Model (phenotype)**	**Anatomical system***
Ccdc85c	108203602-108275425	59.23	hhy-hydrocephalus Intracranial hemorrhage subcortical heterotopia	
Hhipl1	108305918-108328300	59.37		ld, ru
Cyp46a1	108334381-108362234	59.41		hm
Eml1	108370957-108539617	59.46	HeCo-hydrocephalus and subcortical heterotopia	bn, ce, cf, gs, ns, ve,
Evl	108554720-108688516	59.46		ce, cv, bn, em, he, hm, is, np
Degs2	108686792-108702306	59.46		
Gm34220	108790035-108792743		syntenic	
Slc25a29	108825873-108835883	59.62		
Slc25a47	108849813-108856815	59.65		
Wars	108860030-108894174	59.66		he, hm, ve
Yy1	108792973-108816632	59.58		ce, gs, hm, is, np, ns, rs
Wdr25	108894228-109028452	59.7		
Begain	109032187-109068217	59.98		
Dlk1	109452823-109463336	60.17		at, ce, cv, em, gs, he, hm, is, lb, rp, ve

* Mammalian Phenotype Browser (MGI)[3] at=adipose tissue, bn= behavioral/neurological, cv=cardiovascular ce=cellular, cf=craniofacial, em=embryo, gs=growth size, he=hematopoietic, hm= homeostasis/metabolism, is=immune system, ld=limbs/digits, lb=liver/biliary, np= normal phenotype, ns=nervous system, rp=reproductive, rs=respiratory, ru=renal/urinary, ve=vision/eye

References

1 Richards, M. W. *et al.* Crystal structure of EML1 reveals the basis for Hsp90 dependence of oncogenic EML4-ALK by disruption of an atypical beta-propeller domain. *Proc Natl Acad Sci U S A* **111**, 5195-5200, doi:10.1073/pnas.1322892111 (2014).

2 Zerbino, D. R. *et al.* Ensembl 2018. *Nucleic Acids Res* **46**, D754-D761, doi:10.1093/nar/gkx1098 (2018).

3 Law, M. & Shaw, D. R. Mouse Genome Informatics (MGI) Is the International Resource for Information on the Laboratory Mouse. *Methods Mol Biol* **1757**, 141-161, doi:10.1007/978-1-4939-7737-6_7 (2018).
